# Efficient Single-Cell Transgene Induction in *Caenorhabditis elegans* Using a Pulsed Infrared Laser

**DOI:** 10.1534/g3.113.007682

**Published:** 2013-10-01

**Authors:** Matthew A. Churgin, Liping He, John I. Murray, Christopher Fang-Yen

**Affiliations:** * Department of Bioengineering, School of Engineering and Applied Sciences, University of Pennsylvania, Philadelphia, Pennsylvania 19104; † Department of Genetics, Perelman School of Medicine, University of Pennsylvania, Philadelphia, Pennsylvania 19104; ‡ Department of Physics, Korea University, Anam-dong, Seongbuk-gu, Seoul 136-701, South Korea

**Keywords:** transgene induction, heat shock, *C. elegans*

## Abstract

The coupling of transgenes to heat shock promoters is a widely applied method for regulating gene expression. In *C. elegans*, gene induction can be controlled temporally through timing of heat shock and spatially via targeted rescue in heat shock mutants. Here, we present a method for evoking gene expression in arbitrary cells, with single-cell resolution. We use a focused pulsed infrared laser to locally induce a heat shock response in specific cells. Our method builds on and extends a previously reported method using a continuous-wave laser. In our technique, the pulsed laser illumination enables a much higher degree of spatial selectivity because of diffusion of heat between pulses. We apply our method to induce transient and long-term transgene expression in embryonic, larval, and adult cells. Our method allows highly selective spatiotemporal control of transgene expression and is a powerful tool for model organism biology.

Methods for inducing transgene expression in a tissue-specific or cell-specific manner are important for investigating the roles of genes and cells in development, physiology, and behavior. Cell-specific expression has been achieved by use of cell-specific promoters, intersectional promoters, and inducible strategies (Bacaj and Shaham 2007; Wei *et al.* 2012), some of which also enable temporal control of transgene activity. However, these methods depend on cell-specific promoters or promoter combinations, which are not available in many cases. Moreover, these strategies need to be developed for every cell undergoing study, making them impractical for application in many different cell types.

A truly general method would be capable of inducing transgene expression in arbitrary cells. A method called infrared laser–evoked gene operator (IR-LEGO) (Kamei *et al.* 2009) made progress toward this goal. In this technique, a focused continuous-wave (CW) infrared laser beam was used to heat a small volume in the organism. This heating was coupled to transgene expression by using a heat shock–inducible promoter. Although shown to be effective for some cases, this method has two important limitations that have limited its adoption: first, it causes substantial diffusion of heat to neighboring cells, such that off-target transgene induction was difficult or impossible to avoid for small cells; and, second, the efficiency of transgene induction was low for many cell types. Here, we present an improved method for laser-induced heat shock that overcomes these two limitations. First, we used a pulsed rather than CW infrared laser beam to heat targeted cells with dramatically reduced off-target heating. Second, we have optimized conditions to improve the efficiency of transgene induction to as high as 95%, depending on cell type in diverse embryonic and postembryonic cell types.

Theory suggests that pulsed illumination could reduce the size of the heated region as illustrated in Figure 1A. During CW illumination, the temperature rapidly reaches an approximately steady-state (time-independent) distribution. For a small source of heat in an infinite homogenous medium, the steady-state laser-induced temperature increase outside the source decreases as the inverse of the distance to the source. By contrast, a brief pulse of heating at a small source will induce a Gaussian temperature profile that decreases sharply with distance (Supporting Information, File S1). Given equal maximum temperatures at the laser focus, the maximum temperature at any nonzero distance from the laser focus should be smaller for pulsed laser heating than for CW heating.

**Figure 1:**
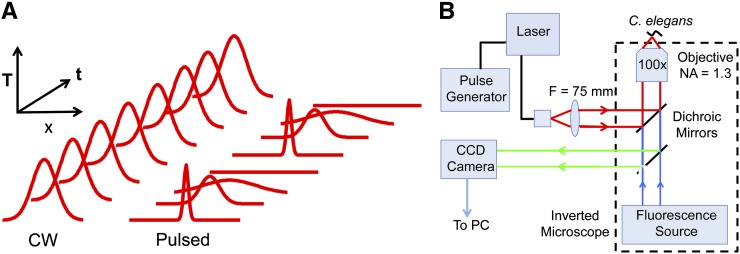
Pulsed and continuous-wave (CW) laser heating. (A) Principle of pulsed-laser-induced gene expression. By allowing time for heat to dissipate after each pulse, a pulsed laser heats a smaller volume than CW illumination, avoids heating neighboring cells, and confines gene induction to the targeted cell. (B) Experimental set-up.

We implemented pulsed heating using an inexpensive, thermoelectrically cooled, fiber-coupled diode laser with a emission wavelength (1480 nm) that is absorbed efficiently by water. The beam emerging from the fiber end was collimated by a lens, reflected from a short-pass dichroic beam splitter, and then entered a 100×, NA 1.3, oil immersion objective that focused the beam onto the sample mounted on an inverted microscope (Figure 1B).

## Results

**Figure 2:**
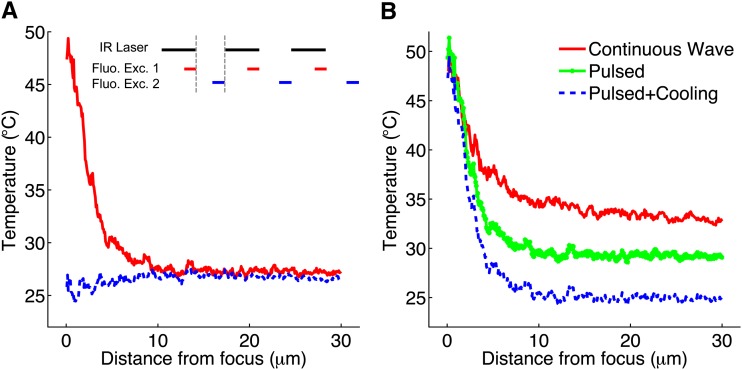
Pulsed laser temperature profile. (A) Spatial heating profile for IR laser pulsed at 1 kHz repetition frequency and 0.2 msec pulse length, as measured using the temperature dependence of GFP. The temperature profile during the IR laser pulse (red) peaks at the laser focus and decays sharply away from the focus. The temperature profile between pulses (blue) is nearly uniform. Inset (not to scale): fluorescence blue LED excitation pulses applied during (red) or after (blue) the PIR laser (black). (B) Comparison of spatial heating profiles for continuous-wave (CW) laser (solid red), pulsed laser (dotted green), and pulsed laser with cooled objective (dashed blue).

We compared the temperature distributions for CW and pulsed infrared irradiation by using GFP-expressing OP50 *E. coli* as a temperature sensor. GFP fluorescence intensity decreases linearly with temperature by approximately 1%/°C over the range of 20–60° (Kamei *et al.* 2009) (Figure S1). We measured the CW laser spatial temperature profile by measuring the spatial patterns of laser-induced change in fluorescence intensity, and we measured the temperature changes during pulsed irradiation by using pulsed blue LED illumination to selectively excite GFP at specific time intervals relative to the infrared laser pulse (Figure 2A). The temperature decays to a nearly uniform distribution when the laser is in the off state, preventing heat build-up away from the laser focus. Modulating the laser power, repetition frequency, and duty cycle (fraction of time on) allowed us to tune the peak temperature and extent of spatial heating (Figure S2 and Figure S3).

Because the laser heating profile is sharply peaked at the laser focus, reducing the overall temperature of the sample served to minimize the size of the region experiencing heat shock. To reduce baseline temperature, we cooled the sample from 23° to 18° by passing cooled water through flexible plastic tubing wrapped around the objective lens, which is in thermal contact with the sample via the immersion oil. We measured the temperature at the sample using a microthermocouple placed inside an agar pad and confirmed that it was equal to the objective temperature to within 0.1°.

Given a hypothetical peak temperature of 50°, we sought to determine over how large a region the laser-induced temperature exceeded 35°, a common temperature used for whole-body heat shock in worms, using the following three illumination modes: CW illumination; pulsed infrared (PIR) illumination; and pulsed illumination with objective cooling. The radius of the region heated above 35° decreased from 12 μm under CW illumination to 5 μm under PIR and to 3 μm under PIR with objective cooling (Figure 2B). Previous work established that the extent of axial heating was approximately twice that of lateral heating (Kamei *et al.* 2009), suggesting that the axial resolution of pulsed illumination with cooling is approximately 6 μm. The four-fold reduction in lateral and axial heated radius corresponds to a 64-fold reduction in heated volume compared to CW illumination.

We confirmed that our data from *E. coli* accurately represented temperature shifts in *C. elegans* by measuring temperature changes in single GFP-expressing mechanosensory neurons in intact worms (Figure S4).

**Figure 3:**
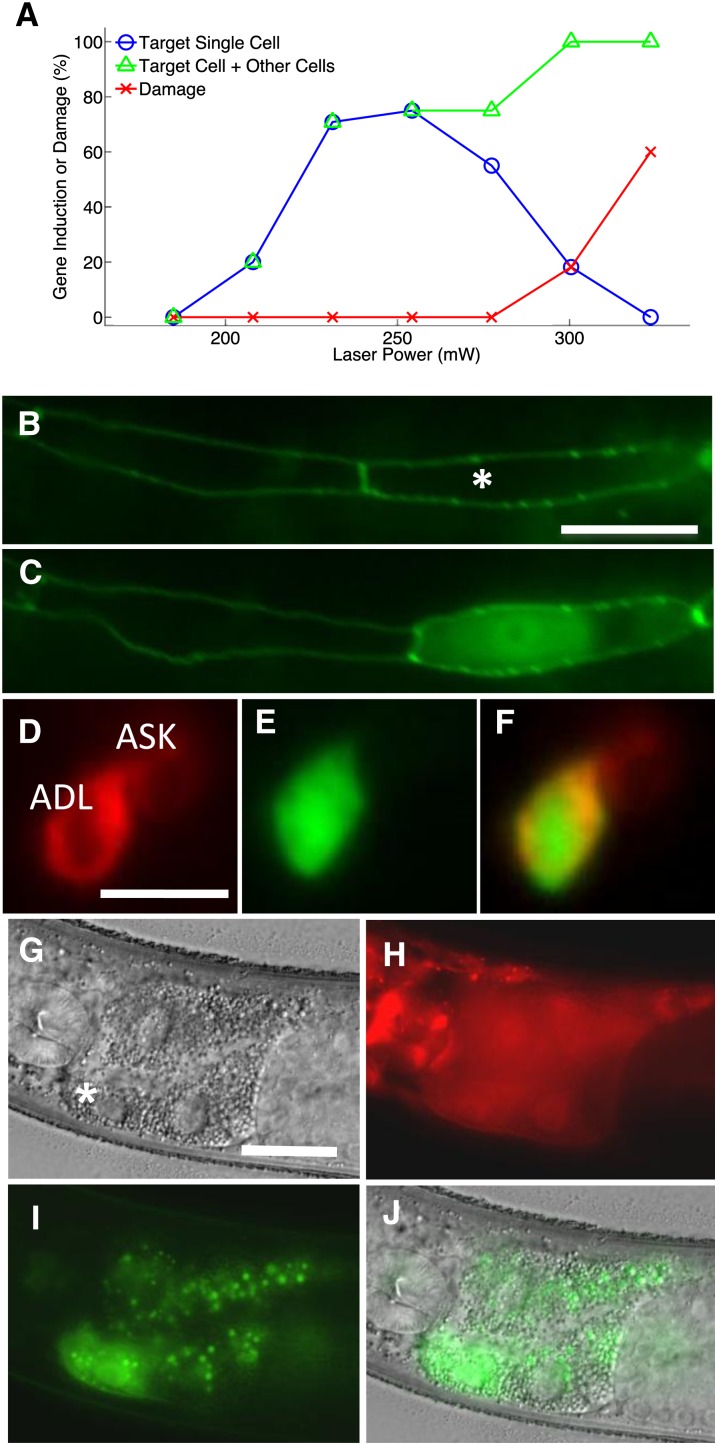
Efficient pulsed laser–induced gene expression in *C. elegans*. (A) Gene induction and damage rate plotted against laser power for seam cells in L4 animals. Blue circles represent gene expression in only the targeted cell. Green triangles indicate expression in the target cell and other cells. Red crosses indicate cell damage. n > 10 for each point. (B) Two seam cells in an L4 animal before laser heat shock. White asterisk indicates the targeted cell. Scale bar, 10 μm. (C) The same two seam cells 3 hr after heat shock. Cytoplasmic GFP is visible in only the targeted cell. (D) Single-cell GFP expression in neurons of L2 larvae. Red lipophilic DiI stains the cell membranes of amphid neurons. ADL was targeted for heat shock. Scale bar, 5 μm. (E) At 3 hr after heat shock, cytoplasmic GFP is visible in only ADL. (F) Merge of (D) and (E). (G) FLP-coupled gene expression in a single intestinal cell (white asterisk). Scale bar, 20 μm. (H) FRT-flanked mCherry expression. (I) GFP induction in the targeted cell. (J) Merge of (G) and (I).

We next sought to determine whether the reduced spatial extent of pulsed laser–induced heating translated to more selective transgene induction compared with CW illumination. We first quantified the induction efficiency of a PIR laser in inducing a heat shock response in the seam cells of L4 larvae. Seam cells are specialized hypodermal cells 20 μm in length located beneath the cuticle (skin) of the worm. We identified these cells with a GFP-tagged adherens junction marker *ajm-1*::GFP. Heat shock activation was assayed by scoring fluorescence 3 hr after laser heating of worms expressing GFP under the control of the *hsp16.2* promoter (Figure 3, B and C). Our irradiation protocol consisted of a pulse repetition frequency of 30 Hz, pulse length of 1 msec, and duration of 6 min. The objective was cooled from room temperature of 24° to 20°. We found that laser powers between 220 and 260 mW induced GFP expression in 75% of targeted single cells in L4 worms, with no expression visible in untargeted neighbor cells (n = 40). Our peak single-cell gene induction rate of 75% in seam cells using a PIR laser is almost twice that (40%) previously reported for a CW laser (Kamei *et al.* 2009).

To test the limits of spatial selectivity of our method, we targeted head neurons in L2 larvae. The cell bodies of these neurons are among the smallest in the animal, approximately 3–5 μm in diameter. To assist in cell identification, we used the red fluorescent lipophilic dye DiI to stain cell membranes of the amphid (sensory) neurons (Shaham 2006). We then applied the same laser heating protocol as applied to seam cells and assayed cells for GFP expression 3 hr after heat shock. We observed GFP in the targeted neuron, ADL, with no visible expression in neighboring cells or pharyngeal muscle, 30% of the time (Figure 3, D–F, Table S1). We were also able to induce gene expression in the amphid neuron AWB (25.9% single-cell induction rate) (Table S1) and mechanosensory neuron ALM (92.3% single-cell induction rate) (Table S1) without observing off-target expression. These results indicate that our method can be used to induce gene expression in a highly selective manner in the *C. elegans* nervous system.

Next, we used single-cell heat shock to induce transgene modifications through the site-specific recombinase FLP. In contrast to direct heat shock activation, which induces transient expression, expression of FLP, when combined with the appropriate FRT-flanked transgene, is capable of inducing changes such as permanent induction of transgene expression or selective knockout of a rescuing transgene (Davis *et al.* 2008). To test this concept, we expressed FLP under a heat shock promoter and used it to excise a termination cassette upstream of the GFP coding sequence. Single intestinal cells targeted by our pulsed laser displayed robust and permanent GFP expression within 18 hr (66.7% induction, n = 21) (Figure 3, G–J), whereas nontargeted cells did not. Therefore, our method is capable of engineering permanent genetic changes in single cells.

**Figure 4:**
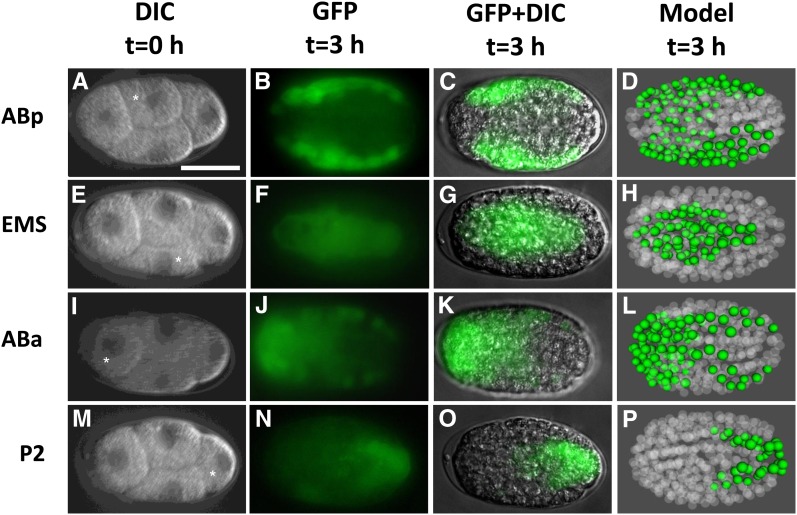
Efficient pulsed laser–induced gene expression in the daughters of individual cells in *C. elegans* embryos. (A) Four-cell-stage embryo just before laser heat shock, with ABp denoted by a white asterisk. (B) Cytoplasmic GFP seen in only the descendants of ABp 3 hr after heat shock. (C) DIC microscopy image merged with (B) to show embryo stage. (D) AceTree simulation of an embryo 3 hr after four-cell stage showing the descendants of ABp (labeled green). The fluorescence pattern in (B) and (C) agree, suggesting only ABp was heat-shocked. The same results as for ABp are shown for targeting EMS (E–H), ABa (I–L), and P2 (M–P). White asterisks indicate the cell targeted for heat shock. All cells were targeted during the four-cell stage for 15 sec with pulse repetition frequency of 100 Hz and pulse length of 2.5 msec. The microscope objective was not cooled during these experiments. Scale bar, 20 μm.

Finally, we tested the ability of our method to induce gene expression in embryonic cells. During early embryonic development, tissue-specific promoters are not active because cell types have not yet been specified. However, the positions and cell types produced by each early embryonic blastomere are predictable because *C. elegans* develops through an invariant lineage (Sulston *et al.* 1983). Induction of transgenes in individual targeted embryonic cells will be useful for tracing cell positions in mutants or for lineage-specific rescue experiments. We individually targeted each of the cells in four-cell-stage embryos in P*hsp-16.2*::GFP worms. With a modified heating protocol, we could both maximize hatch rate (>74%) and observe efficient induction of GFP expression (>80%) in the descendants of ABp, ABa, and EMS (Table S2). The frequency of GFP induction in P2 was lower, perhaps because of the transcriptional quiescence of this cell during the four-cell stage (Seydoux *et al.* 1996), but we still observed expression in this lineage in 26% of embryos. We confirmed that expression was limited to the targeted cell and its progeny by comparing the observed fluorescence pattern 3 hr after heat shock (Figure 4, B, F, J, and N) with an AceTree computer simulation of the expected positions at that stage (Boyle *et al.* 2006) (Figure 4, D, H, L, and P). Our finding that single-cell heat shock is capable of inducing gene expression in the daughters of individual cells during development points to its potential usefulness in genetically manipulating developing embryos with fine spatiotemporal control.

## Discussion

In summary, we have developed a robust, efficient, and specific method for transgene induction in single cells. Our method can evoke gene expression in a variety of cell types while avoiding gene induction in neighbors only approximately 5 μm from the laser focus. This represents a vast improvement in spatial selectivity compared with existing methods. We have optimized irradiation protocols for efficiently inducing expression without visible damage in both developing embryos and larvae. Our method is inexpensive and similar in technical complexity to laser ablation systems, which are used widely in studies of *C. elegans* (Fang-Yen *et al.* 2011), and therefore should extend readily to other transparent organisms such as embryos and larvae of *Drosophila* and zebrafish. We expect our method will be particularly useful in studying development, in which expression of transgenes with fine spatiotemporal control can help elucidate the cellular and molecular mechanisms governing cell fate decisions (Abdus-Saboor *et al.* 2011), body patterning (Pearson *et al.* 2005), and organogenesis (Mango 2009).

## Methods

### Laser and microscope

We combined a fiber-coupled infrared diode laser (Fitel FOL1425RUZ-317, wavelength 1480 nm, maximum power 400 mW) with an inverted microscope (Nikon TE2000-S) equipped with DIC and fluorescence optics and with sample stage raised using a height adjustment kit (T-BSUK). The laser was controlled by a diode laser driver (Opto Power OPC-PS03-A) and a thermoelectric cooler driver (Thorlabs TEC2000). The emission from the fiber tip was collimated with a plano-convex lens (focal length f = 75 mm). A short-pass dichroic mirror (Thorlabs DMSP1000R) positioned between the objective lens and the fluorescence turret reflected the collimated laser beam into the back of the objective lens (Nikon Plan Fluor 100×, NA 1.3). A pulse generator (Stanford Research Systems DG535) connected to the laser driver was used to control the frequency, duration, and number of laser pulses. We recorded images using a CCD camera (Photometrics Cascade 1k) and Micro-Manager software. Data analysis was performed using MATLAB (Mathworks Corp.).

The infrared beam was not detectable directly by our CCD. To determine the location of the laser focus, we used laser trapping of 1-μm-diameter polystyrene beads. Each day, before experiments with worms, we used our set-up to illuminate a 0.25% suspension of 1 μm polystyrene beads (Polysciences) mounted between a glass slide and coverslip. We marked the location of a trapped bead on the computer monitor; this mark corresponded to the location of the laser focus and was used as a guide to perform laser heating of cells.

To lower the temperature of the sample, we wrapped plastic tubing (Tygon) around the microscope objective. Water at 5° was run through the tubing by a temperature-controlled water circulator (Brookfield). The circulator was turned on at least 1 hr before laser heat shock to allow the temperature of the objective to stabilize. We periodically monitored the temperature of the objective using a thermocouple.

### Temperature calibration of GFP

GFP-expressing OP50 *E. coli* were spread onto a 10% agarose pad using a platinum wire pick and the slide containing the pad was placed on a temperature-controllable microscope stage. The temperature was controlled with a water circulator. We inserted a microthermocouple (Physitemp) into the agarose pad to monitor the temperature of the pad. Fluorescence images of the same field of view were taken periodically as the temperature was shifted. Fluorescent illumination was only applied during image acquisition and the exposure time per image was less than 5 sec. Images were corrected for photobleaching. The mean value of the same ROI was plotted *vs.* temperature as measured by the thermocouple (Figure S1).

### Temperature measurement using GFP

As in the temperature calibration experiments, we used GFP-expressing *E. coli* on a 10% agarose pad. For CW temperature measurements, the laser power was adjusted to the desired setting and two fluorescence images of the same field of view were acquired. The first image was acquired with the CW laser disabled and the second image was acquired with CW laser enabled. The exposure time was the same for both images but varied day to day from 0.5 to 2 sec. We calculated the laser spatial heating profile by dividing the pixel intensity values between the two images and converting the decrease in fluorescence intensity during laser heating into temperature change using the calibration described (Figure S1).

For pulsed laser temperature measurements, we used a 10-W blue LED (center wavelength 445 nm). The LED light was collimated with a 40-mm focal length aspheric condenser lens (diameter = 50 mm) and directed through the fluorescence port of the modified inverted microscope. The pulse generator controlling the laser was configured to also control the LED driver via a solid-state relay (Grayhill 70-ODC5).

To measure the temperature changes as the laser pulsed at 1 kHz with 0.2-msec pulse length (Figure 2A), the blue LED was set to illuminate from 0.1 to 0.2 msec during the pulse cycle (Figure 2A inset). In this case, pulsing the LED in phase with the pulsed laser made the camera detect GFP fluorescence only during the laser on state. As with CW temperature measurements, two images were taken: one with the pulsed laser disabled and one with the pulsed laser enabled. Both images were acquired with the same exposure time, which varied day to day from 1 to 10 sec. Next, the LED was set to illuminate during 0.8 to 0.9 msec of the pulse cycle and two images were acquired in the same manner as before. In this case, pulsing the LED out of phase with the pulsed laser made the camera detect GFP fluorescence only during the off-state of the laser (Figure 2A). The pulsed laser spatial heating profiles during the laser on state and off state were determined in the same way as for CW temperature measurements using the GFP temperature calibration to convert fluorescence decrease into temperature shift (Figure S1).

We sought to confirm that the *in vitro* scenario accurately described *in vivo* temperature shifts. We conducted an experiment using live immobilized P*mec-7*::*GFP* worms expressing GFP in mechanosensory neurons as a temperature sensor, analogous to the *E. coli* experiments. We targeted the cell body of one of the bilateral ALM neurons and measured peak temperature as a function of IR laser power. We found no difference in peak temperature between *E. coli* and the ALM cell body proximal to the coverslip (Figure S4). However, targeting ALM distal to the coverslip generated a slightly higher peak temperature, possibly because of removal of a heat-sinking effect of the glass coverslip. Our results indicate that the *in vitro* model of laser heating accurately describes *in vivo* temperature shifts.

### Strains

Worms were cultured on NGM agar seeded with OP50 bacteria using standard methods (Sulston and Hodgkin 1988). Larval and adult worms were handled with a platinum wire pick. Embryos were handled by mouth pipette. Worms used for larvae and adult experiments were grown at 20°. Worms used for embryo experiments were grown at 15°. All heat shock experiments except those using FLP recombinase were performed using the strain ST66 (*ncIs17* [P*hsp16.2*::EGFP]). We occasionally observed GFP expression in single cells before heat shock. All GFP induction experiments were performed using cells in which GFP expression was not observed before heat shock. To monitor *in vivo* temperature shifts in adult worms, we used strain *muIs32* (P*mec-7*::*gfp*). To identify and target seam cells for heat shock, we constructed the strain YX40 by crossing *ncIs17* and *ncIs13*. The transgene *ncIs13* contained P*ajm-1*::*GFP*, an adherens junction marker that delineated seam cells in larval worms. GFP was never visible in seam cells before heat shock. Experiments in which the neuron ALM was targeted for heat shock were performed using the strain containing *muIs32*; *ncIs18* [P*hsp16.2*::mRFP]. RFP expression was never visible in ALM before heat shock. All experiments involving amphid neurons and all experiments involving embryos were performed using the strain containing transgene *ncIs17*. GFP expression was never visible in amphid neurons or embryos before heat shock. For experiments using FLP, we constructed the strain YX29 (*qhEx15* [P*hsp16.48*::FLP; P*his-72*::FRTmCherryFRT_GFP::*unc-54* 3′UTR]). One of four of the first intestinal ring cells in L3 or L4 worms was heated with 25% duty cycle at 100 Hz for 3 min. GFP expression was scored 18–24 hr after heat shock.

For seam cell heat shock experiments, L4 animals were removed from food for 1 d before. After heat shock, animals were recovered to a new seeded OP50 plate. We found that this process increased the efficiency of single-cell heat shock activation four-fold in addition to increasing the damage threshold (Figure 3A, Figure S5), perhaps via a heat shock preconditioning effect (Kourtis *et al.* 2012). Animals were only starved for use in experiments in which seam cells were targeted. It is possible that starvation would increase efficiency in other cell types. To label the cell membranes of amphid neurons (Figure 3, D and E), we used DiI staining (Shaham 2006).

### Larvae laser heat shock and mounting

During imaging and heating, 5 to 10 worms were immobilized at a time using 10% agarose pads and 0.1 μm polystyrene beads (Kim *et al.* 2013). Worms underwent the pulsed laser heat shock and were recovered to a seeded NGM agar plate within 1 hr of mounting.

To activate FLP-coupled heat shock–mediated gene expression in L3 and L4 animals (Figure 3, G–J), intestinal cells were targeted with the IR laser for 3 min with pulse repetition frequency of 100 Hz and pulse length of 2.5 msec. GFP induction was scored 18–48 hr after heat shock.

### Embryo laser heat shock and mounting

For embryo experiments, four-cell-stage embryos were mounted as previously described (Bao and Murray 2011). All slides contained control embryos not subjected to laser illumination. Embryos were imaged and heat-shocked within 20 min of mounting. Cells were targeted during the four-cell stage for 15 sec with pulse repetition frequency of 100 Hz and pulse length of 2.5 msec. At 2–3 hr after laser irradiation, we scored GFP induction using a Leica DM2500 fluorescence microscope. Embryos remained mounted overnight in a humidified chamber to score hatching the next morning (Table S2). The hatch rate for control animals was 100%.

### Computer simulation

We used AceTree (Boyle *et al.* 2006) to generate 3D images of the expected positions for the progeny of each four-cell blastomere after 3 hr, based on a reference model of embryogenesis (Richards *et al.* 2013) (Figure 4, D, H, L, and P).

## Supplementary Material

Supporting Information

## References

[bib1] Abdus-Saboor I. Mancuso V. P. Murray J. I. Palozola K. Norris C., 2011 Notch and Ras promote sequential steps of excretory tube development in C. elegans. Development 138: 3545–3555 21771815 10.1242/dev.068148 PMC3143567

[bib2] Bacaj T. Shaham S., 2007 Temporal control of cell-specific transgene expression in Caenorhabditis elegans. Genetics 176: 2651–2655 17603102 10.1534/genetics.107.074369 PMC1950662

[bib3] Bao, Z., and J. I. Murray, 2011 Mounting Caenorhabditis elegans embryos for live imaging of embryogenesis. Cold Spring Harbor Protoc. 10.1101/pdb.prot065599 10.1101/pdb.prot065599 21880814

[bib4] Boyle T. J. Bao Z. Murray J. I. Araya C. L. Waterston R. H., 2006 AceTree: a tool for visual analysis of Caenorhabditis elegans embryogenesis. BMC Bioinformatics 7: 275 16740163 10.1186/1471-2105-7-275 PMC1501046

[bib5] Davis M. W. Morton J. J. Carroll D. Jorgensen E. M., 2008 Gene activation using FLP recombinase in C. elegans. PLoS Genet. 4: e1000028 18369447 10.1371/journal.pgen.1000028 PMC2265415

[bib6] Fang-Yen C. Gabel C. Bargmann C. I. Samuel A. D. T. Avery L. 2011 Laser microsurgery in C. elegans, pp. 177–206 in *Caenorhabditis elegans: Modern Biological Analysis of an Organism*, *Methods in Cell Biology*, edited by J. H. Rothman and A. Singson Elsevier Academic Press 10.1016/B978-0-12-394620-1.00006-0 PMC3617498 22226524

[bib7] Kamei Y. Suzuki M. Watanabe K. Fujimori K. Kawasaki T., 2009 Infrared laser-mediated gene induction in targeted single cells in vivo. Nat. Methods 6: 79–81 19079252 10.1038/nmeth.1278

[bib8] Kim E. Sun L. Gabel C. V. Fang-Yen C., 2013 Long-term imaging of Caenorhabditis elegans using nanoparticle-mediated immobilization. PLoS ONE 8: e53419 23301069 10.1371/journal.pone.0053419 PMC3536676

[bib9] Kourtis N. Nikoletopoulou V. Tavernarakis N., 2012 Small heat-shock proteins protect from heat-stroke-associated neurodegeneration. Nature 490: 213–218 22972192 10.1038/nature11417

[bib10] Mango S. E., 2009 The molecular basis of organ formation: insights from the C. elegans foregut. Annu. Rev. Cell Dev. Biol. 25: 597–628 19575642 10.1146/annurev.cellbio.24.110707.175411 PMC4882101

[bib11] Pearson J. C. Lemons D. McGinnis W., 2005 Modulating Hox gene functions during animal body patterning. Nat. Rev. Genet. 6: 893–904 16341070 10.1038/nrg1726

[bib12] Richards J. L. Zacharias A. L. Walton T. Burdick J. T. Murray J. I., 2013 A quantitative model of normal Caenorhabditis elegans embryogenesis and its disruption after stress. Dev. Biol. 374: 12–23 23220655 10.1016/j.ydbio.2012.11.034 PMC3548946

[bib13] Seydoux G. Mello C. C. Pettitt J. Wood W. B. Priess J. R., 1996 Repression of gene expression in the embryonic germ lineage of C. elegans. Nature 382: 713–716 8751441 10.1038/382713a0

[bib14] Shaham S., 2006 Methods in cell biology. WormBook. http://www.wormbook.org. /10.1895/wormbook.1.49.1.

[bib15] Sulston J. E. Schierenberg E. White J. G. Thomson J. N., 1983 The embryonic cell lineage of the nematode Caenorhabditis elegans. Dev. Biol. 100: 64–119 6684600 10.1016/0012-1606(83)90201-4

[bib16] Sulston, J., and J. Hodgkin, 1988 Methods, pp. 587–606 in *The Nematode C. elegans*, edited by W. B. Wood. Cold Spring Harbor Laboratory Press, Cold Spring Harbor Laboratory, New York.

[bib17] Wei X. Potter C. J. Luo L. Shen K., 2012 Controlling gene expression with the Q repressible binary expression system in Caenorhabditis elegans. Nat. Methods 9: 391–395 22406855 10.1038/nmeth.1929 PMC3846601

