# Supplementary material for: Efficient Single-Cell Transgene Induction in Caenorhabditis elegans Using a Pulsed Infrared Laser
Source: G3 (Bethesda). 2013 Oct 1;3(10):1827–32. doi: 10.1534/g3.113.007682 (PMC3789807; doi:10.1534/g3.113.007682)
Supplement: Supporting Information [file supp_3_10_1827__index.html]

Efficient Single-Cell Transgene Induction in Caenorhabditis elegans Using a Pulsed Infrared Laser — Supporting Information 

# Efficient Single-Cell Transgene Induction in *Caenorhabditis elegans* Using a Pulsed Infrared Laser

## Supporting Information for Churgin *et al.*, 2013

**Files in this Data Supplement:**

- Supporting Information - Figures S1-S5, File S1, and Tables S1-S2 (PDF, 755 KB)
- Figure S1 - Temperature Dependence of GFP-expressing *E. coli* (PDF, 529 KB)
- Figure S2 - Frequency Modulates Baseline Temperature and Width of Spatial Temperature Distribution (PDF, 518 KB)
- Figure S3 - Laser Power and Frequency Influence Peak Temperature (PDF, 412 KB)
- Figure S4 - Comparison of *in vitro* and *in vivo* temperature shift (PDF, 527 KB)
- Figure S5 - Starvation Increases Probability of Single Cell Gene Expression and Increases Damage Threshold (PDF, 400 KB)
- File S1 - Equations describing heat transport during continuous-wave and pulsed laser illumination (PDF, 530 KB)
- Table S1 - Gene expression rate for single neurons targeted during L2 stage (PDF, 396 KB)
- Table S2 - Gene expression and hatch rate for single cells targeted during four-cell stage (PDF, 397 KB)
