# Supplementary material for: Efficient Single-Cell Transgene Induction in Caenorhabditis elegans Using a Pulsed Infrared Laser
Source: G3 (Bethesda). 2013 Oct 1;3(10):1827–32. doi: 10.1534/g3.113.007682 (PMC3789807; doi:10.1534/g3.113.007682)
Supplement: Supporting Information [file supp_g3.113.007682_007682SI.pdf]

G3 · Genes | Genomes | Genetics

Efficient single-cell transgene induction in *Caenorhabditis elegans* using a pulsed infrared laser

Matthew A. Churgin[*], Liping He[*], John I. Murray[§], and Christopher Fang-Yen[*,**]

*Department of Bioengineering, School of Engineering and Applied Sciences, University of Pennsylvania, Philadelphia PA 19104, USA.

§ Department of Genetics, Perelman School of Medicine, University of Pennsylvania, Philadelphia PA 19104, USA

****** Department of Physics, Korea University, Anam-dong, Seongbuk-gu, Seoul 136-701, S. Korea

DOI: 10.1534/g3.113.007682

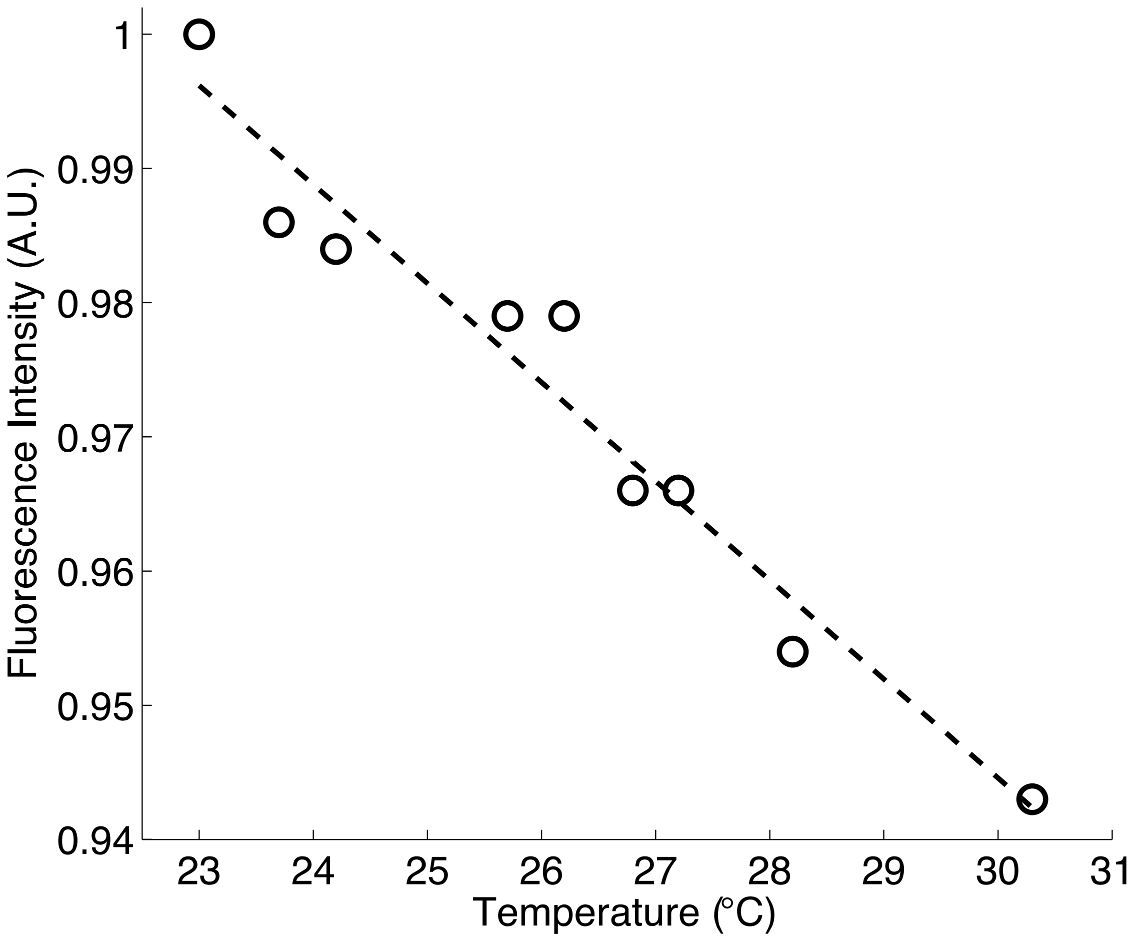

Figure S1 Temperature Dependence of GFP-expressing *E. coli*. Fluorescence intensity of GFP (solid black curve) expressed in *E. coli* measured on a thermo-controllable stage decreases linearly. In all calibration experiments we used the fit line, F, to convert changes in fluorescence intensity into temperature changes. The fit (dashed black curve) obeys f = -0.0079t + 1.18, where t is the temperature in °C and f is the fluorescence intensity relative to the fluorescence intensity at 23 °C. The fluorescence temperature dependence measured here is comparable to that previously described (Kamei et al. 2009).

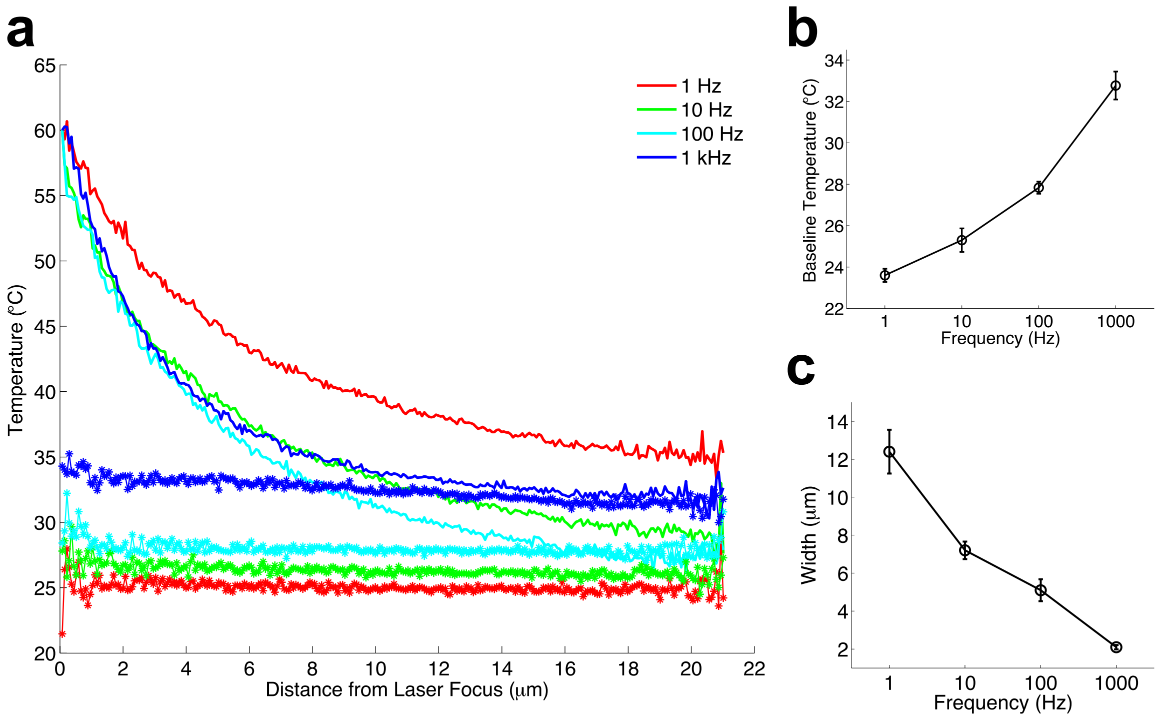

Figure S2 Frequency Modulates Baseline Temperature and Width of Spatial Temperature Distribution
The pulse repetition frequency and pulse length modulate the baseline temperature and width of the spatial temperature distribution, with higher repetition frequencies raising the temperature throughout the field of view and shorter pulses narrowing the temperature distribution. Experiments were performed without sample cooling, and the laser power was adjusted to maintain peak temperature within 5° C between different repetition frequencies. (**a**) Peak (solid curves) and baseline (asterisks) temperature distribution are shown for 4 different frequencies with a 10% laser duty cycle. The width of the temperature distribution decreases with frequency (see part **b**) and the baseline temperature increases with frequency (see part **c**).

(**b**) Temperature at the laser focus during the laser off state increases with frequency. For a given duty cycle (10%) and laser power, the temperature at the laser focus during the laser off state increased with the pulse repetition frequency. Error bars represent SEM for 3 measurements.

(**c**) Spatial width of temperature decay curve decreases with frequency. Width was defined as the radial distance from the laser focus (peak of the temperature distribution) to the point where the temperature equaled the baseline temperature plus 25% of the difference between the peak and baseline temperatures. Error bars represent SEM for 3 measurements.

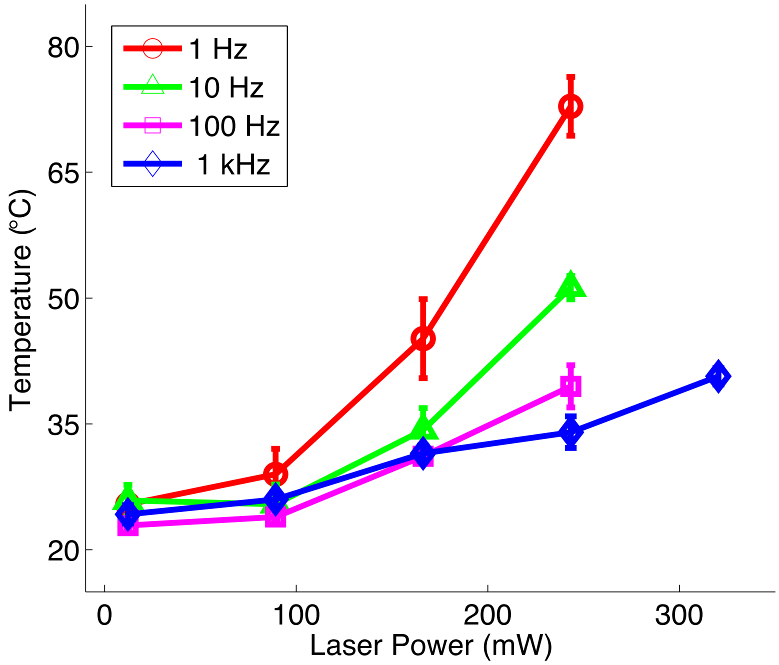

Figure S3 Laser Power and Frequency Influence Peak Temperature
The temperature at the laser focus during the pulsed laser on state was measured for a range of laser powers and repetition frequencies while holding the duty cycle at 10%. Temperature curves increase approximately linearly with laser power. As expected, higher frequencies have shallower slopes than lower frequencies, owing to the fact that as frequency increases the laser is on for less continuous time and as a result lower peak temperatures are generated. Error bars represent SEM for 3 measurements.

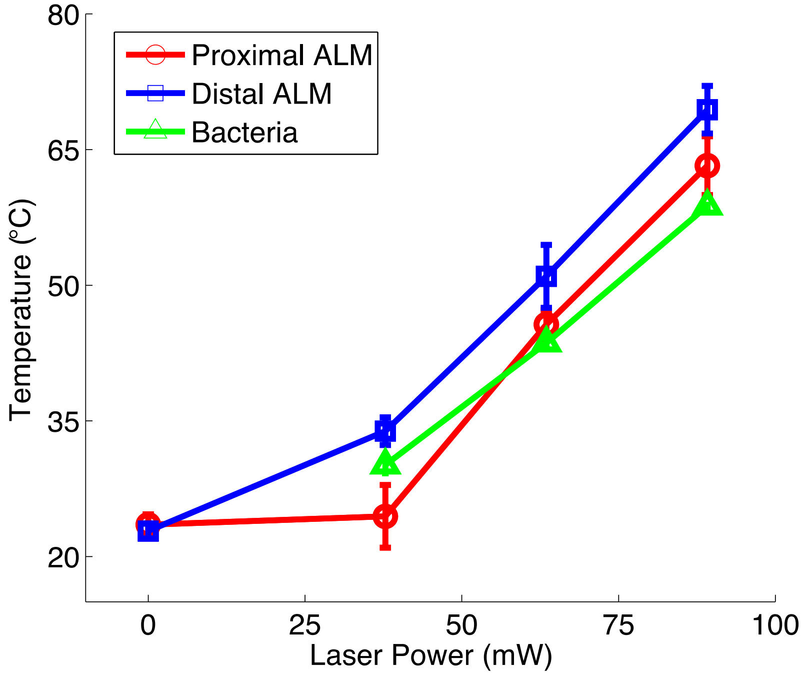

Figure S4 Comparison of *in vitro* and *in vivo* temperature shift.
The temperature shift was calculated for a continuous wave laser in GFP-expressing *E. coli* (green). *In vivo* temperature shifts were measured using the transgene *mec-7::gfp* which is expressed in the bilateral ALM neurons. Adult hermaphrodites were immobilized and the ALM neurons were targeted with the CW laser. When immobilized, the ALM neurons lie either adjacent (proximal) to the coverslip or adjacent to the mounting pad (distal to the coverslip). Targeting the proximal ALM cell body generated a similar temperature curve as targeting GFP-expressing *E. coli*. Targeting the distal ALM generated slightly higher (5 °C) temperature shifts that we attribute to a heat sinking effect of the glass coverslip. Error bars represent SEM for 3 measurements.

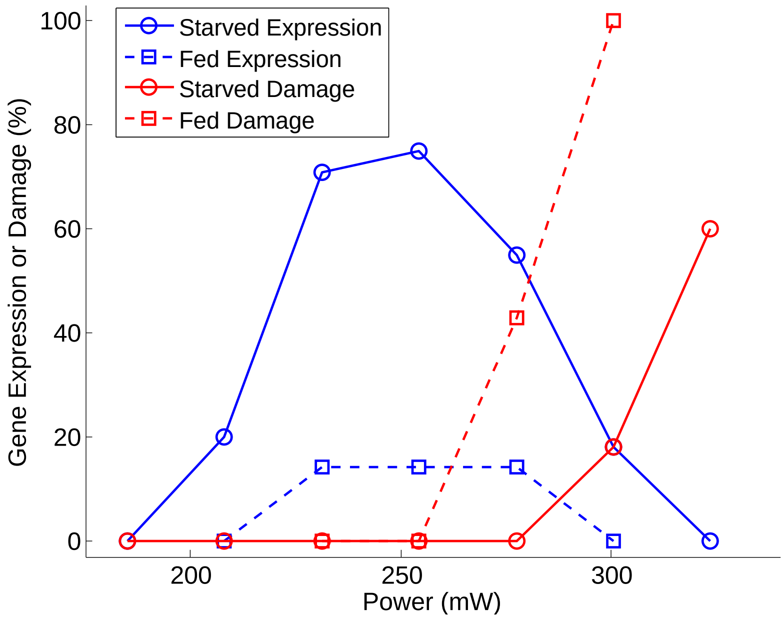

Figure S5 Starvation Increases Probability of Single Cell Gene Expression and Increases Damage Threshold
For a range of laser powers we measured GFP induction in seam cells for starved (solid blue curve) and well-fed (dashed blue curve) L4 animals. Starved animals exhibited a much higher rate of GFP induction. Additionally, the rate at which we observed damage seam cells after heat shock was different between starved (solid red curve) and well-fed (dashed red curve) L4 animals. The threshold at which damage became apparent was higher for starved animals then for well-fed animals.

File S1

Equations describing heat transport during continuous-wave and pulsed laser illumination

Heat transport during continuous-wave and pulsed laser illumination
The dynamics of the temperature distribution is described by the heat equation with external input:

$$\rho c_p \frac{\partial T}{\partial t} = \kappa \nabla^2 T + P$$

Where ρ is the density, c_p is the specific heat, κ is the thermal conductivity, and $P(\mathbf{r},t)$ is the heat power deposited by the laser. Suppose $P=0$ outside a small radius r_0.
For **continuous-wave illumination** P is constant. At steady-state (time derivative of temperature is zero) we have

$$T(r,t) = T_0 + \frac{P}{4\pi\kappa r}$$

for $r > r_0$. Therefore the laser-induced temperature shift decreases as the inverse of the radius from the center of the heated region.
For **pulsed illumination**, an infinitely small and short pulse of heat creates a thermal distribution described by the fundamental solution

$$T(r,t) = T_0 + \frac{E}{4\pi\rho c_p(\alpha t)^{3/2}} e^{-r^2/4\alpha t}$$

where

$$\alpha = \frac{\kappa}{\rho c_p}$$

is the thermal diffusivity and E is the total energy deposited by the pulse:

$$E = \int_{\text{pulse}} P dt$$

Therefore in pulsed illumination the laser-induced temperature shift decreases exponentially with the square of the distance to the heat source.

Table S1 Gene expression rate for single neurons targeted during L2 stage

Cell Targeted	Single cell induction rate	Single cell + other cell induction rate
ADL	9/30 (30%)	12/30 (40%)
AWB	7/27 (25.9%)	10/27 (37%)
ALM	12/13 (92.3%)	0/13 (0%)

Table S2 **Gene expression and hatch rate for single cells targeted during four-cell stage**

Cell Targeted	GFP induction rate	Hatch rate
ABp	30/31 (96.8%)	23/31 (74.2%)
EMS	20/25 (80%)	23/25 (92.0%)
ABa	21/26 (80.8%)	20/26 (76.9%)
P2	7/27 (25.9%)	23/27 (85.2%)
