# Supplementary material for: Efficient Single-Cell Transgene Induction in Caenorhabditis elegans Using a Pulsed Infrared Laser
Source: G3 (Bethesda). 2013 Oct 1;3(10):1827–32. doi: 10.1534/g3.113.007682 (PMC3789807; doi:10.1534/g3.113.007682)
Supplement: Supporting Information [file supp_g3.113.007682_FigureS1.pdf]

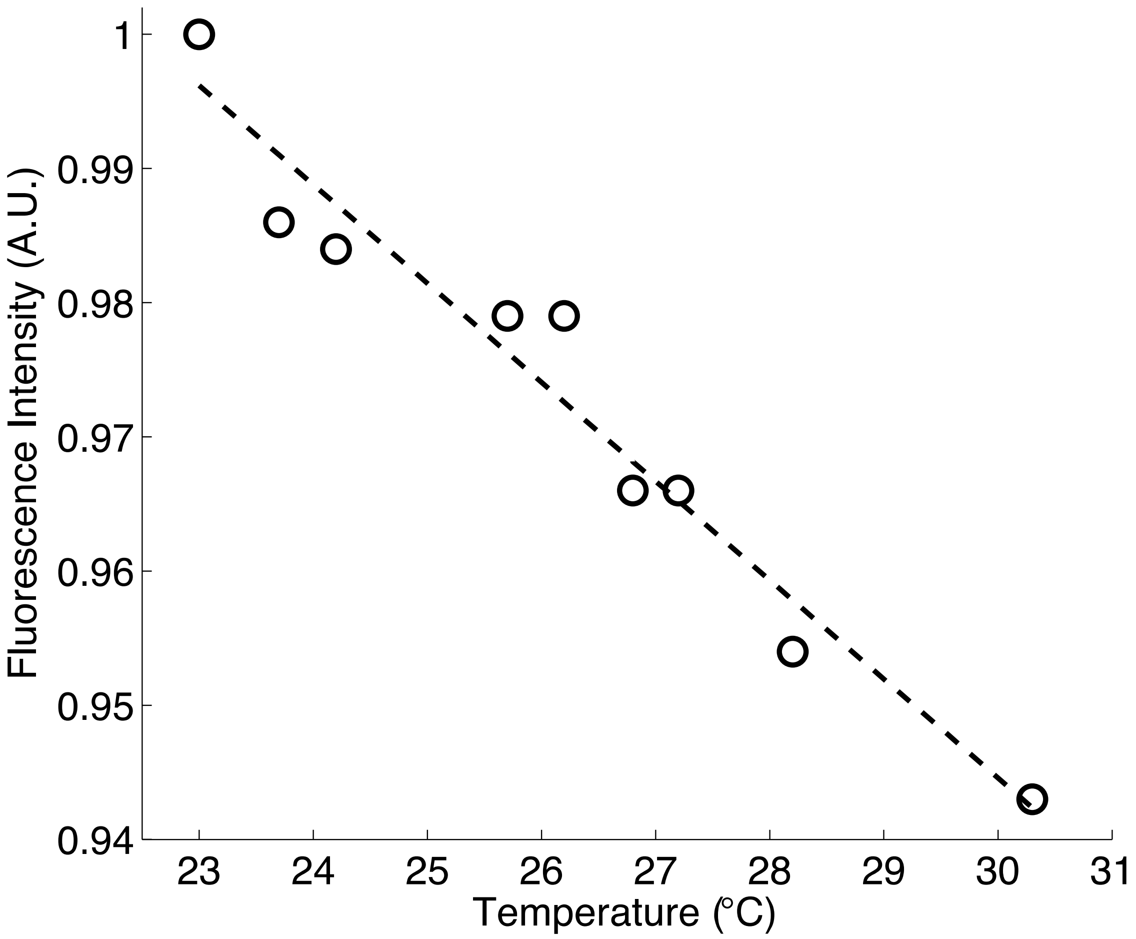

Figure S1 Temperature Dependence of GFP-expressing *E. coli.* Fluorescence intensity of GFP (solid black curve) expressed in *E. coli* measured on a thermo-controllable stage decreases linearly. In all calibration experiments we used the fit line, F, to convert changes in fluorescence intensity into temperature changes. The fit (dashed black curve) obeys f = -0.0079t + 1.18, where t is the temperature in °C and f is the fluorescence intensity relative to the fluorescence intensity at 23 °C. The fluorescence temperature dependence measured here is comparable to that previously described (Kamei et al. 2009).
