# Supplementary material for: Efficient Single-Cell Transgene Induction in Caenorhabditis elegans Using a Pulsed Infrared Laser
Source: G3 (Bethesda). 2013 Oct 1;3(10):1827–32. doi: 10.1534/g3.113.007682 (PMC3789807; doi:10.1534/g3.113.007682)
Supplement: Supporting Information [file supp_g3.113.007682_FigureS2.pdf]

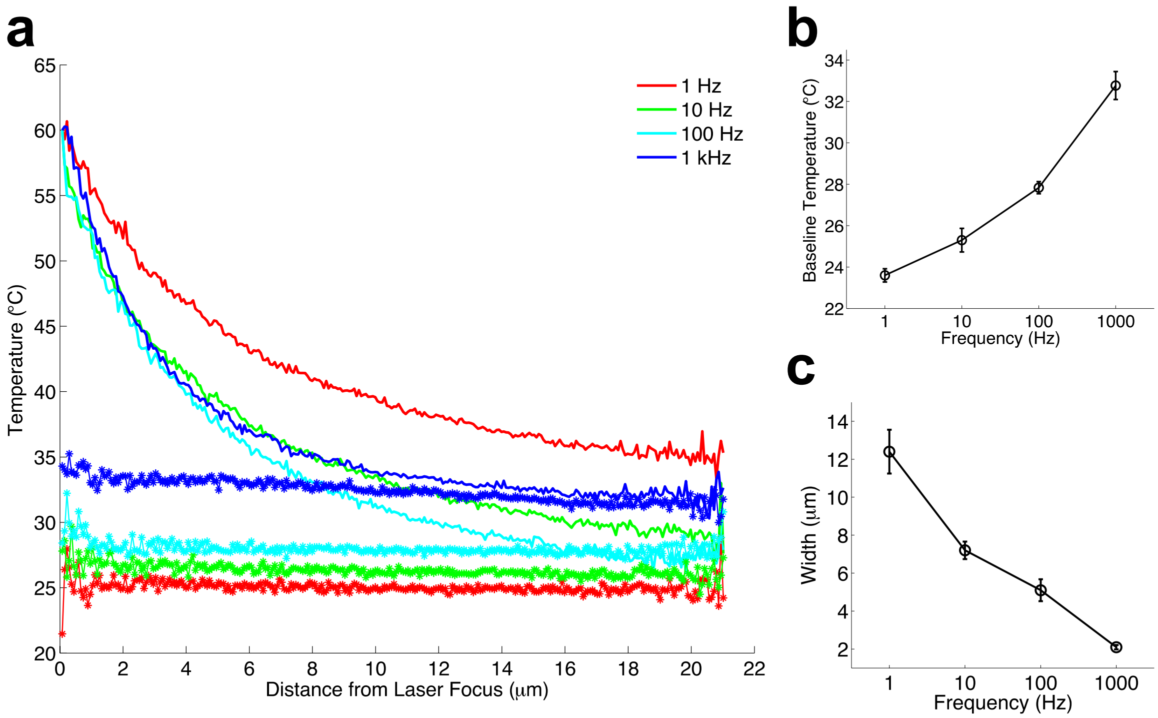

Figure S2 Frequency Modulates Baseline Temperature and Width of Spatial Temperature Distribution
The pulse repetition frequency and pulse length modulate the baseline temperature and width of the spatial temperature distribution, with higher repetition frequencies raising the temperature throughout the field of view and shorter pulses narrowing the temperature distribution. Experiments were performed without sample cooling, and the laser power was adjusted to maintain peak temperature within 5° C between different repetition frequencies. (**a**) Peak (solid curves) and baseline (asterisks) temperature distribution are shown for 4 different frequencies with a 10% laser duty cycle. The width of the temperature distribution decreases with frequency (see part **b**) and the baseline temperature increases with frequency (see part **c**).
(**b**) Temperature at the laser focus during the laser off state increases with frequency. For a given duty cycle (10%) and laser power, the temperature at the laser focus during the laser off state increased with the pulse repetition frequency. Error bars represent SEM for 3 measurements.
(**c**) Spatial width of temperature decay curve decreases with frequency. Width was defined as the radial distance from the laser focus (peak of the temperature distribution) to the point where the temperature equaled the baseline temperature plus 25% of the difference between the peak and baseline temperatures. Error bars represent SEM for 3 measurements.
