# Supplementary material for: Efficient Single-Cell Transgene Induction in Caenorhabditis elegans Using a Pulsed Infrared Laser
Source: G3 (Bethesda). 2013 Oct 1;3(10):1827–32. doi: 10.1534/g3.113.007682 (PMC3789807; doi:10.1534/g3.113.007682)
Supplement: Supporting Information [file supp_g3.113.007682_FigureS3.pdf]

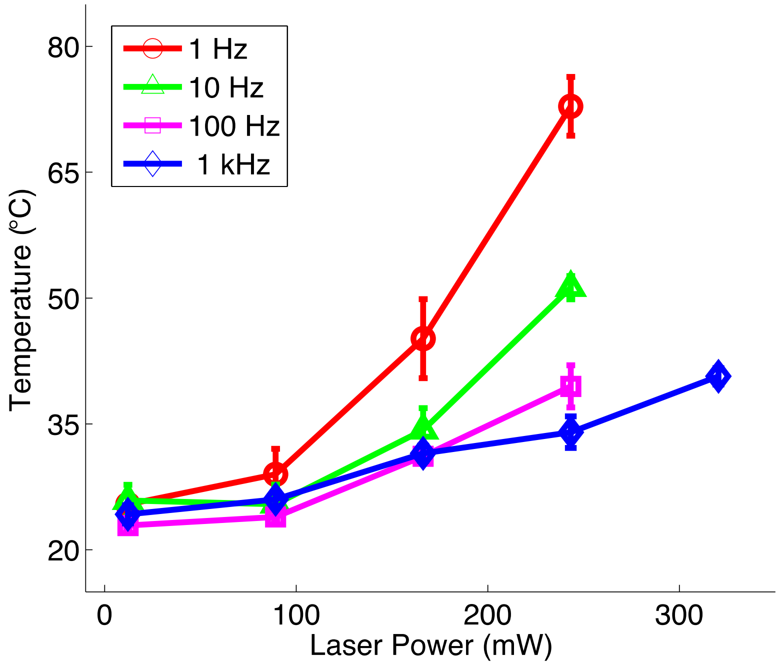

Figure S3 Laser Power and Frequency Influence Peak Temperature
The temperature at the laser focus during the pulsed laser on state was measured for a range of laser powers and repetition frequencies while holding the duty cycle at 10%. Temperature curves increase approximately linearly with laser power. As expected, higher frequencies have shallower slopes than lower frequencies, owing to the fact that as frequency increases the laser is on for less continuous time and as a result lower peak temperatures are generated. Error bars represent SEM for 3 measurements.
