# Supplementary material for: Efficient Single-Cell Transgene Induction in Caenorhabditis elegans Using a Pulsed Infrared Laser
Source: G3 (Bethesda). 2013 Oct 1;3(10):1827–32. doi: 10.1534/g3.113.007682 (PMC3789807; doi:10.1534/g3.113.007682)
Supplement: Supporting Information [file supp_g3.113.007682_FigureS4.pdf]

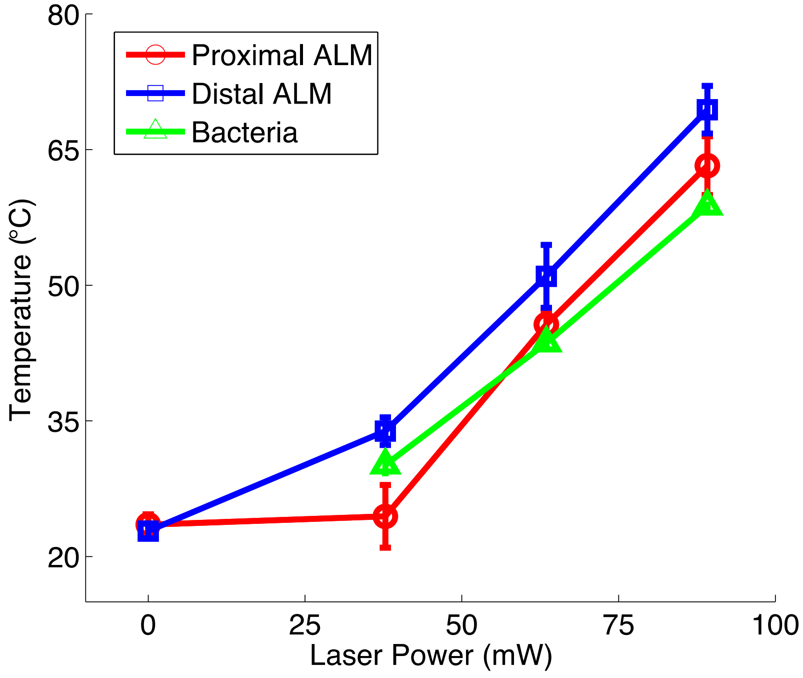

Figure S4 Comparison of *in vitro* and *in vivo* temperature shift.
The temperature shift was calculated for a continuous wave laser in GFP-expressing *E. coli* (green). *In vivo* temperature shifts were measured using the transgene *mec-7::gfp* which is expressed in the bilateral ALM neurons. Adult hermaphrodites were immobilized and the ALM neurons were targeted with the CW laser. When immobilized, the ALM neurons lie either adjacent (proximal) to the coverslip or adjacent to the mounting pad (distal to the coverslip). Targeting the proximal ALM cell body generated a similar temperature curve as targeting GFP-expressing *E. coli*. Targeting the distal ALM generated slightly higher (5 °C) temperature shifts that we attribute to a heat sinking effect of the glass coverslip. Error bars represent SEM for 3 measurements.
