# Supplementary material for: Efficient Single-Cell Transgene Induction in Caenorhabditis elegans Using a Pulsed Infrared Laser
Source: G3 (Bethesda). 2013 Oct 1;3(10):1827–32. doi: 10.1534/g3.113.007682 (PMC3789807; doi:10.1534/g3.113.007682)
Supplement: Supporting Information [file supp_g3.113.007682_FigureS5.pdf]

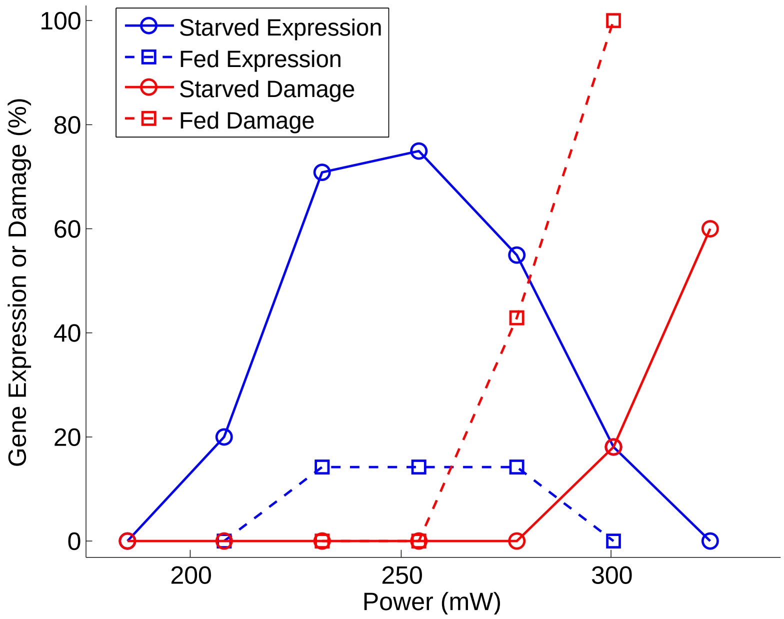

Figure S5 Starvation Increases Probability of Single Cell Gene Expression and Increases Damage Threshold
For a range of laser powers we measured GFP induction in seam cells for starved (solid blue curve) and well-fed (dashed blue curve) L4 animals. Starved animals exhibited a much higher rate of GFP induction. Additionally, the rate at which we observed damage seam cells after heat shock was different between starved (solid red curve) and well-fed (dashed red curve) L4 animals. The threshold at which damage became apparent was higher for starved animals then for well-fed animals.
