# Supplementary material for: Efficient Single-Cell Transgene Induction in Caenorhabditis elegans Using a Pulsed Infrared Laser
Source: G3 (Bethesda). 2013 Oct 1;3(10):1827–32. doi: 10.1534/g3.113.007682 (PMC3789807; doi:10.1534/g3.113.007682)
Supplement: Supporting Information [file supp_g3.113.007682_FileS1.pdf]

File S1

Equations describing heat transport during continuous-wave and pulsed laser illumination

Heat transport during continuous-wave and pulsed laser illumination
The dynamics of the temperature distribution is described by the heat equation with external input:

$$\rho c_p \frac{\partial T}{\partial t} = \kappa \nabla^2 T + P$$

Where ρ is the density, c_p is the specific heat, κ is the thermal conductivity, and $P(\mathbf{r},t)$ is the heat power deposited by the laser. Suppose $P=0$ outside a small radius r_0.
For **continuous-wave illumination** P is constant. At steady-state (time derivative of temperature is zero) we have

$$T(r,t) = T_0 + \frac{P}{4\pi\kappa r}$$

for $r > r_0$. Therefore the laser-induced temperature shift decreases as the inverse of the radius from the center of the heated region.
For **pulsed illumination**, an infinitely small and short pulse of heat creates a thermal distribution described by the fundamental solution

$$T(r,t) = T_0 + \frac{E}{4\pi\rho c_p(\alpha t)^{3/2}} e^{-r^2/4\alpha t}$$

where

$$\alpha = \frac{\kappa}{\rho c_p}$$

is the thermal diffusivity and E is the total energy deposited by the pulse:

$$E = \int_{\text{pulse}} P dt$$

Therefore in pulsed illumination the laser-induced temperature shift decreases exponentially with the square of the distance to the heat source.
