# Supplementary material for: Efficient Single-Cell Transgene Induction in Caenorhabditis elegans Using a Pulsed Infrared Laser
Source: G3 (Bethesda). 2013 Oct 1;3(10):1827–32. doi: 10.1534/g3.113.007682 (PMC3789807; doi:10.1534/g3.113.007682)
Supplement: Supporting Information [file supp_g3.113.007682_TableS1.pdf]

Table S1 Gene expression rate for single neurons targeted during L2 stage

Cell Targeted	Single cell induction rate	Single cell + other cell induction rate
ADL	9/30 (30%)	12/30 (40%)
AWB	7/27 (25.9%)	10/27 (37%)
ALM	12/13 (92.3%)	0/13 (0%)
