# Supplementary material for: Efficient Single-Cell Transgene Induction in Caenorhabditis elegans Using a Pulsed Infrared Laser
Source: G3 (Bethesda). 2013 Oct 1;3(10):1827–32. doi: 10.1534/g3.113.007682 (PMC3789807; doi:10.1534/g3.113.007682)
Supplement: Supporting Information [file supp_g3.113.007682_TableS2.pdf]

Table S2 Gene expression and hatch rate for single cells targeted during four-cell stage

Cell Targeted	GFP induction rate	Hatch rate
ABp	30/31 (96.8%)	23/31 (74.2%)
EMS	20/25 (80%)	23/25 (92.0%)
ABa	21/26 (80.8%)	20/26 (76.9%)
P2	7/27 (25.9%)	23/27 (85.2%)
